# Association of Neural and Emotional Impacts of Reward Prediction Errors With Major Depression

**DOI:** 10.1001/jamapsychiatry.2017.1713

**Published:** 2017-08-02

**Authors:** Robb B. Rutledge, Michael Moutoussis, Peter Smittenaar, Peter Zeidman, Tanja Taylor, Louise Hrynkiewicz, Jordan Lam, Nikolina Skandali, Jenifer Z. Siegel, Olga T. Ousdal, Gita Prabhu, Peter Dayan, Peter Fonagy, Raymond J. Dolan

**Affiliations:** 1 Max Planck University College London Centre for Computational Psychiatry and Ageing Research, London, England; 2 Wellcome Trust Centre for Neuroimaging, University College London, London, England; 3 Department of Radiology, Haukeland University Hospital, Bergen, Norway; 4 Gatsby Computational Neuroscience Unit, University College London, London, England; 5 Developmental Neuroscience Unit, Anna Freud Centre, London, England

## Abstract

**Importance:**

Major depressive disorder (MDD) is associated with deficits in representing reward prediction errors (RPEs), which are the difference between experienced and predicted reward. Reward prediction errors underlie learning of values in reinforcement learning models, are represented by phasic dopamine release, and are known to affect momentary mood.

**Objective:**

To combine functional neuroimaging, computational modeling, and smartphone-based large-scale data collection to test, in the absence of learning-related concerns, the hypothesis that depression attenuates the impact of RPEs.

**Design, Setting, and Participants:**

Functional magnetic resonance imaging (fMRI) data were collected on 32 individuals with moderate MDD and 20 control participants who performed a probabilistic reward task. A risky decision task with repeated happiness ratings as a measure of momentary mood was also tested in the laboratory in 74 participants and with a smartphone-based platform in 1833 participants. The study was conducted from November 20, 2012, to February 17, 2015.

**Main Outcomes and Measures:**

Blood oxygen level–dependent activity was measured in ventral striatum, a dopamine target area known to represent RPEs. Momentary mood was measured during risky decision making.

**Results:**

Of the 52 fMRI participants (mean [SD] age, 34.0 [9.1] years), 30 (58%) were women and 32 had MDD. Of the 74 participants in the laboratory risky decision task (mean age, 34.2 [10.3] years), 44 (59%) were women and 54 had MDD. Of the smartphone group, 543 (30%) had a depression history and 1290 (70%) had no depression history; 918 (50%) were women, and 593 (32%) were younger than 30 years. Contrary to previous results in reinforcement learning tasks, individuals with moderate depression showed intact RPE signals in ventral striatum (*z* = 3.16; *P* = .002) that did not differ significantly from controls (*z* = 0.91; *P* = .36). Symptom severity correlated with baseline mood parameters in laboratory (ρ = −0.54; *P* < 1 × 10^−6^) and smartphone (ρ = −0.30; *P* < 1 × 10^−39^) data. However, participants with depression showed an intact association between RPEs and happiness in a computational model of momentary mood dynamics (*z* = 4.55; *P* < .001) that was not attenuated compared with controls (*z* = −0.42; *P* = .67).

**Conclusions and Relevance:**

The neural and emotional impact of RPEs is intact in major depression. These results suggest that depression does not affect the expression of dopaminergic RPEs and that attenuated RPEs in previous reports may reflect downstream effects more closely related to aberrant behavior. The correlation between symptom severity and baseline mood parameters supports an association between depression and momentary mood fluctuations during cognitive tasks. These results demonstrate a potential for smartphones in large-scale computational phenotyping, which is a goal for computational psychiatry.

## Key Points

**Question:**

Is the neural and emotional impact of reward prediction errors attenuated in major depression?

**Findings:**

In a neuroimaging study, depression was not associated with a reduced neural impact of reward prediction errors in a nonlearning context. Depression also was not associated with a reduced emotional impact of reward prediction errors in a laboratory behavioral study and in a smartphone study with 1833 participants.

**Meaning:**

In moderate major depression, impacts of reward prediction errors that are linked to dopamine, known to be attenuated in a learning context, are intact in nonlearning tasks.

## Teaser

This cohort study evaluates reward prediction errors in patients with major depressive disorder vs those without depression using functional neuroimaging, computational modeling, and smartphone-based data.

## Introduction

Major depressive disorder (MDD) is now the leading determinant of years lived with disability worldwide.^1^ The lifetime prevalence of mood disorders is higher than 20% in the United States.^2^ Depression is associated with impaired reward and emotion processing,^3^ and empirical evidence suggests aberrant functioning of the brain’s reward circuitry, specifically within dopaminergic inputs to ventral striatum.^4^ Neuroimaging studies report reduced ventral striatal activity for both anticipation and receipt of rewards in adults^7^ and adolescents^11^ with depression.

Dopaminergic inputs to ventral striatum represent reward prediction errors (RPEs), which are the difference between experienced and predicted rewards.^12^ The RPE signals provide a mechanism for modifying synapses in a manner consistent with reinforcement learning algorithms.^14^ When a decision outcome exceeds expectations, the value associated with the chosen option is increased, making it more likely to be chosen again. Because of their central role in adaptive behavior, understanding how RPE signals are affected by depression is important in explaining aberrant behavior in depressed individuals.

We focus on an a priori region of interest—ventral striatum—that consistently shows attenuated RPEs in depression during reinforcement learning,^15^ supporting prominent hypotheses of reduced dopamine signals in depression.^4^ However, because depression is associated with learning deficits,^17^ another possibility is that reward-processing anomalies are specific to learning. It remains unknown whether depression reduces RPE signals in tasks without a significant learning requirement. Ventral striatum represents RPEs in nonlearning tasks,^19^ and dopamine measurements in this area represent RPEs.^13^ Dopamine release increases ventral striatal blood oxygen level–dependent (BOLD) activity,^20^ while pharmacologic manipulation of dopamine modulates ventral striatal RPE signals.^21^ We tested the specific hypothesis that depression attenuates ventral striatal RPE signals in a task without a significant learning component.

Previous studies suggest that depression also attenuates emotional reactivity.^22^ In healthy individuals, variation in RPEs quantitatively explains momentary mood fluctuations,^23^ while manipulating dopamine affects the association between rewards and momentary mood.^24^ These findings raise the question as to whether depression reduces the emotional impact of RPEs in the absence of learning.

We used a combination of functional magnetic resonance imaging (fMRI) and computational modeling to test the hypothesis that depression is associated with a reduction in neural and emotional impacts of RPEs in a nonlearning context. We also tested this hypothesis in a large-scale study in which we deployed a smartphone-based platform^25^ to obtain a sample larger (n = 1833) than feasible in the laboratory.

## Methods

### Participants

The study was conducted from November 20, 2012, to February 17, 2015. Participants in the laboratory study were recruited from primary medical and psychological care services. We sought participants receiving treatment based on a primary diagnosis of MDD and deemed clinically appropriate for treatment delivery within a primary care setting. This broad group excluded more severe forms of depression managed in secondary care. All participants in the group with depression had MDD with at least 1 moderate-to-severe depressive episode without any psychotic features. Both groups were matched for age, sex, and educational level (Table). We excluded individuals with psychotic, bipolar, and neurologic disorders as well as those with any other psychiatric primary diagnosis, including any anxiety disorders. We also excluded individuals with any diagnosed drug- or alcohol-related disorder. A stepped professional approach was applied, from treating physician to study psychiatrist, to ensure a representative sample. The Hamilton Scale for Depression (HAM-D) was the primary measure of depression severity, and all depressed participants in the fMRI sample had a HAM-D score of at least 14 (a conventional definition of moderate severity).^28^ Participants also completed the Patient Health Questionnaire (PHQ).^29^

**Table.** Differences Between MDD and Control Groups

Characteristic	MDD Behavior (n = 54)	MDD fMRI Only (n = 32)	Control (n = 20)	Statistical Test^a^	*P* Value
Women, No. (%)	34 (63)	20 (63)	10 (50)	Fisher exact	.40
Age, mean (SD), y	34.3 (11.1)	34.1 (9.7)	34.0 (8.3)	*t*_50_ = 0.02	.98
Educational level, mean (SD), y	16.3 (2.2)	16.3 (2.4)	16.4 (1.9)	*t*_50_ = −0.31	.76
HAM-D score, mean (SD)	15.6 (4.1)	16.6 (2.5)	0.6 (1.0)	*t*_50_ = 27.4	<.001
PHQ score, mean (SD)	15.8 (4.7)	16.9 (3.6)	1.1 (1.7)	*t*_50_ = 18.3	<.001
Medication, No. (%)^b^	33 (61)	23 (72)	0	Fisher exact	<.001

Abbreviations: fMRI, functional magenetic resonance imaging; HAM-D, Hamilton Scale for Depression; MDD, major depressive disorder; PHQ, Patient Health Questionnaire.

^a^ Statistical tests compared depressed fMRI and healthy control samples.

^b^ Antidepressant medications included bupropion hydrochloride (1 participant), citalopram hydrobromide (10), fluoxetine hydrochloride (8), mirtazapine (3), nortriptyline hydrochloride (1), quetiapine fumarate (1), sertraline hydrochloride (8), and venlafaxine (2). Only 1 participant with MDD was taking more than 1 antidepressant (mirtazapine and quetiapine).

The study was approved by the National Research Ethics Service Committee for City and East London. All participants gave written informed consent. Participants were compensated with a flat fee in addition to earnings from the tasks described below. 

Exclusion criteria specifically for the fMRI study included claustrophobia and left-handedness in addition to standard MRI safety exclusion criteria (eg, metal implants). Thirty-five depressed and 20 control participants completed the probabilistic reward task in the MRI scanner. There was no significant difference in average performance between groups (choice to observation lottery: 96% for depressed cohort, 97% for control cohort; *z* = 0.26; *P* = .80). To ensure similar behavioral data for neural analyses, we excluded from further analysis 3 depressed participants who failed to choose the observation lottery in more than 30 trials. All participants in the fMRI study also completed the risky decision task, as did an additional 19 depressed participants.

Participants in the smartphone study gave informed consent. The study was approved by the Research Ethics Committee of University College London. Participants were anonymous and not compensated for participation (eMethods in the Supplement). The second edition of the Beck Depression Inventory (BDI-II) questionnaires were completed by 1833 individuals who previously played the risky decision task (eTable in the Supplement). Of these participants, 918 (50%) were women, 593 (32%) were younger than 30 years, 543 (30%) had a history of depression, and 1290 (70%) had no history of depression.

### Procedures

#### Probabilistic Reward Task

The probabilistic reward task was devoid of any learning requirement. The task was specifically designed to ensure a similar level of performance in depressed and control participants in terms of accuracy, reaction time, and earnings.^19^ In each of 164 trials completed in the MRI scanner, participants chose between 2 lotteries and were then shown the outcome of the chosen lottery (eFigure 1 and eMethods in the Supplement).

#### Risky Decision Task

In each trial, participants made choices between safe and risky options (eFigure 2A in the Supplement). Risky options were monetary gambles with 2 potential outcomes. All choice outcomes counted for real money. After every 2 to 3 trials, participants were asked, “How happy are you at this moment?” and moved a cursor along a line to record their current subjective state. Participants completed 160 choice trials and 66 ratings. Chosen outcomes were resolved after a brief delay in half of the gamble choices. In the other half of the choices, the text “outcome added to total” was displayed. We also collected data in this task using a smartphone app, The Great Brain Experiment (http://www.thegreatbrainexperiment.com; available free for iOS and Android operating systems). The app features 8 cognitive science tasks (including “What makes me happy?”) that replicate known laboratory findings.^23^ Participants completed 30 choice trials and 12 ratings. Risky options were represented by spinners with equal probabilities for 2 potential outcomes, and chosen gambles were resolved immediately (eFigure 2B in the Supplement). Participants started with an endowment of 500 points and tried to earn as many points as possible.

#### fMRI Imaging

We recorded BOLD responses during the probabilistic reward task using a 3T MRI scanner (3T Magnetom Trio; Siemens Healthcare) and a 32-channel head coil. Whole-brain T2*-weighted echo-planar imaging data were acquired using a sequence designed to minimize dropout in the striatum, frontal cortex, and amygdala.^32^ Physiological monitoring included measurements of pulse and breathing. Preprocessing and analysis of the echo-planar imaging data were performed using statistical parametric mapping (SPM8; Wellcome Trust Centre for Neuroimaging) following standard procedures (eMethods in the Supplement).

#### Computational Modeling of Momentary Mood

We fitted an established computational model^23^ in which certain rewards (CRs) are chosen instead of a gamble, expected values (EVs) are the average return of chosen gambles, and RPEs resulting from those expectations all exert influences on happiness:
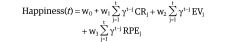
where t and j are trial numbers, w_0_ is a baseline mood parameter, other weights (w) capture influences of different event types, and 0≤γ≤1 is a forgetting factor that makes more recent events more influential than events in earlier trials with an exponential decay. Terms for unchosen options were set to zero, and the RPE was set to zero when the outcome was not revealed. We used happiness as a proxy for what we refer to as momentary mood and related these momentary assessments to clinical measures that capture mood on longer time scales. We used a Bayesian model comparison to validate the model, testing alternate models that omit expectations or split RPE terms into their separate components (eMethods in the Supplement).

### Statistical Analysis

Nonparametric statistical tests that do not assume data are normally distributed were used. These included Wilcoxon signed rank and rank sum tests and Spearman correlation coefficients (ρ). Statistical tests were always performed on continuous variables when available. Performing statistical tests on dichotomized continuous data can lead to artifactual findings.^33^ We also computed correlations after regressing out sex, educational level (having a university degree), and age. All *P* values are 2-tailed. Significance was set at *P* < .05.

## Results

### Probabilistic Reward Task

**Figure 1.**
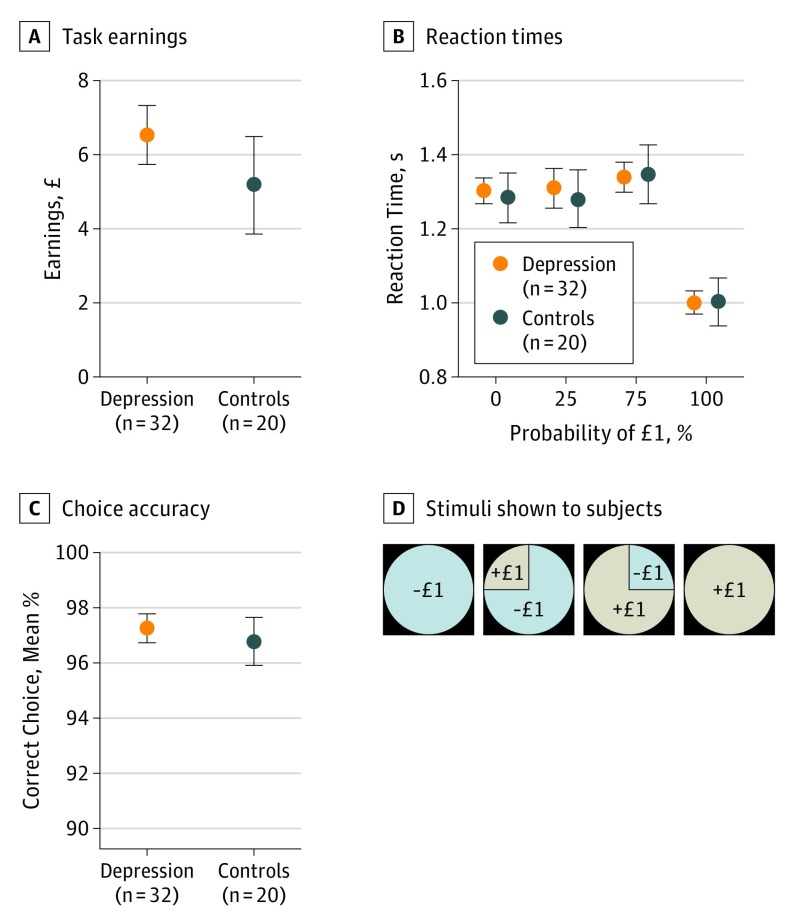
Behavioral Measures for Neuroimaging Experiment Participants played a probabilistic reward task during simultaneous functional magnetic resonance imaging for earnings. In each trial, participants had to select 1 of 2 lotteries and were then shown the outcome. A, Task earnings were similar in depressed and control groups. The task design was such that participants should always choose 1 of 4 observation lotteries with outcomes of gaining or losing £1 (US $1.29) and a probability of 0%, 25%, 75%, or 100% of receiving the better outcome. B, Reaction times were similar in depressed and control groups and faster for certain wins. C, Choice accuracy to the observation lotteries (D) was similar in depressed and control groups. Error bars indicate SEM.

**Figure 2.**
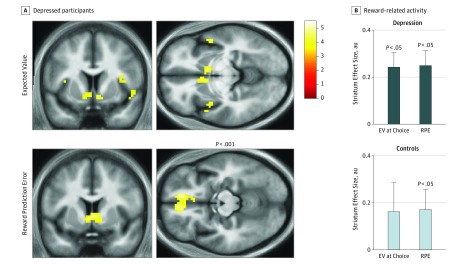
Main Effects of Reward-Related Neural Responses A, Depressed participants (n = 32) showed intact expected value (EV) signals in ventral striatum and intact reward prediction error (RPE) signals in ventral striatum and medial prefrontal cortex. Images are displayed at uncorrected *P* < .001. B, EV and RPE signals in the ventral striatum region of interest were not significantly different in the depressed (n = 32) and control groups (n = 20). Scale indicates *t* statistics; Error bars, SEM; AU, arbitrary units.

We analyzed data in the probabilistic reward task (eFigure 1 in the Supplement) for depressed and control groups that had similar earnings (Figure 1A; mean, £6.02 [US $7.75]; *z* = 1.19, *P* = .24), median reaction times (Figure 1B; mean, 1.26 seconds; *z* = 0.16, *P* = .87), and choice accuracy (Figure 1C; mean, 97%; *z* = 0.28, *P* = .78). Both groups responded faster for certain gains relative to other observation lotteries. During the choice period, BOLD activity in ventral striatum in depressed participants was correlated with the EV of chosen gambles (Figure 2A). During the outcome period, BOLD activity in ventral striatum in depressed participants correlated with predicted RPE (Figure 2A). BOLD activity within a bilateral ventral striatum region of interest (eMethods in the Supplement) was significant in depressed participants for both EV and RPE (both *z* = 3.16, both *P* = .002) (Figure 2B). Furthermore, this activity did not differ significantly for EV (*z* = 0.86, *P* = .39) or RPE (*z* = 0.91, *P* = .36) in depressed participants compared with controls. The RPE parameters were uncorrelated with earnings (ρ = −0.17, *P* = .22), number of errors (ρ = 0.15, *P* = .28), and reaction times (ρ = −0.01, *P* = .95).

The RPEs are the difference between experienced and predicted rewards, and ventral striatal BOLD activity in the entire sample was positively correlated with reward magnitude (*z* = 2.93, *P* = .003) and negatively correlated with lottery EV (*z* = 1.96, *P* = .05), consistent with previous results.^19^ There were no significant differences between the depressed and control groups in reward magnitude (*z* = 1.33, *P* = .18) or EV (*z* = 0.31, *P* = .76) parameters. We also found no significant difference in ventral striatal RPEs between medicated and nonmedicated depressed participants (*z* = 1.43, *P* = .15). The RPE parameters were uncorrelated with symptom severity within the individuals with depression (HAM-D, ρ = −0.08, *P* = .65; PHQ, ρ = 0.06, *P* = .73). Because anhedonia has been linked to attenuated reward impact,^17^ we specifically examined the PHQ scale anhedonia question. Participants with depression exhibited higher anhedonia ratings (mean, 2.2 vs 0.1; *z* = 6.18; *P* < 1 × 10^−9^), but there was no association between anhedonia and ventral striatal RPEs (ρ = 0.13, *P* = .35).

### Risky Decision Task

**Figure 3.**
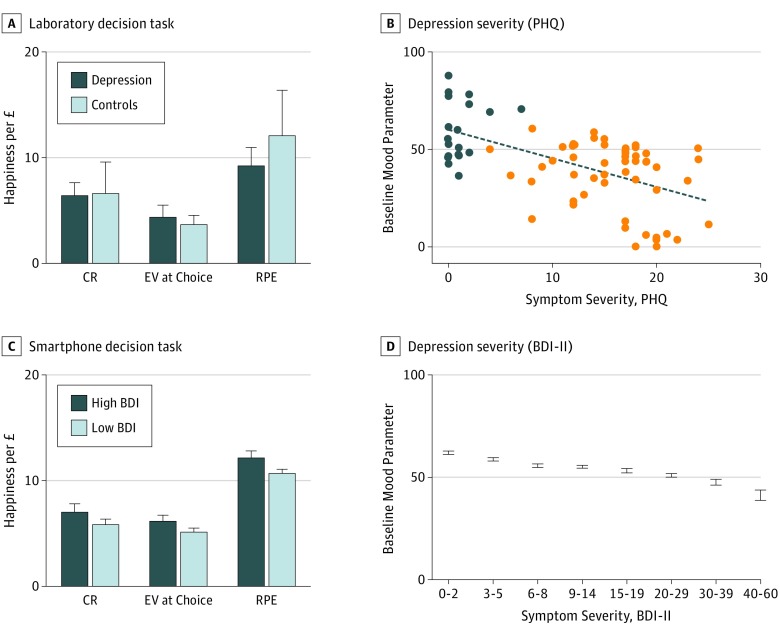
Computational Model of Momentary Mood in Laboratory and Smartphone Experiments A, Participants (depressed, 54; control, 20) played a risky decision task in the laboratory and made a choice on every trial between safe and risky options; they then were shown the gamble expected values (EVs). After every 2 to 3 trials, participants were asked, “How happy are you at this moment?” Parameter weights for past certain rewards (CRs), gamble EVs, and reward prediction errors (RPEs) in the momentary mood computational model were significantly positive in both the depressed and control groups. B, Baseline mood parameters were negatively correlated with symptom severity (Patient Health Questionnaire [PHQ], ρ = −0.54, *P* < 1 × 10^−6^; Hamilton Scale for Depression [HAM-D], ρ = −0.50, *P* < 1 × 10^−5^). C, Participants (n = 1833) played a similar risky decision task on their smartphones and also completed the Beck Depression Inventory second edition (BDI-II) questionnaire. Parameter weights were not reduced for participants with high (≥15) compared with low (<15) BDI-II scores. The impact of RPEs was not reduced in depression and was greater in participants with worse symptom severity (ρ = 0.05, *P* = .01). D, Baseline mood parameters were negatively correlated with symptom severity (BDI-II, ρ = −0.30, *P* < 1 × 10^−39^). Error bars indicate SEM.

We analyzed data from the laboratory sample (n = 74) and found no significant difference between depressed and control groups in reaction times (*z* = 0.85, *P* = .40), earnings (*z* = 1.22, *P* = .22), or risk taking (*z* = 1.36, *P* = .17). The momentary mood computational model accounted for happiness ratings similarly in the depressed (mean *r*^2^ = 0.35) (eFigure 2C in the Supplement) and control groups (mean *r*^2^ = 0.33). Our model was preferred by Bayesian model comparison to alternative models (eResults in the Supplement). Replicating prior studies,^23^ parameter weights for past CRs, EVs, and RPEs were positive in controls (all *z*>2.40, *P* < .05) (Figure 3A). Parameter weights were also positive in depressed participants (CR, *z* = 5.50; *P* < .001; EV, *z* = 4.01, *P* < .001; RPE, *z* = 4.55, *P* < .001) and did not differ significantly from controls (CR, *z* = 0.06, *P* = .96; EV, *z* = 0.64, *P* = .53; RPE, *z* = −0.42, *P* = .67). There was no reduction in the emotional impact of RPEs with increasing symptom severity (PHQ, ρ = −0.13, *P* = .25; HAM-D, ρ = −0.15, *P* = .19). However, after accounting for momentary mood dynamics, baseline mood parameters (w_0_ in the computational model) were negatively correlated with symptom severity (PHQ, ρ = −0.54, *P* < 1 × 10^−6^; HAM-D, ρ = −0.50, *P* < 1 × 10^−5^) (Figure 3B).

In the smartphone sample (n = 1833) (eFigure 2B in the Supplement), we found the momentary mood computational model again accounted for happiness ratings (mean *r*^2^ = 0.63). There was a modest increase in the quality of model fits (ρ = 0.05, *P* = .02) with depression severity. The forgetting factor was not related to the BDI-II in the smartphone sample (ρ = 0.03, *P* = .16) and was similar in the depressed and control groups in the laboratory sample (γ = 0.34, *z* = 1.37, *P* = .17). The emotional impact of RPEs, if anything, increased with symptom severity (ρ = 0.05, *P* = .01) (Figure 3C). This association was present in individuals who had never received antidepressant medications (n = 1301; ρ = 0.07, *P* = .01) and after regressing out age, educational level, and sex effects (ρ = 0.05, *P* = .03). Baseline mood parameters (estimated while simultaneously accounting for mood dynamics due to expectations and RPEs) were negatively correlated with symptom severity (BDI-II, ρ = −0.30, *P* < 1 × 10^−39^) (Figure 3D). This association was present in individuals who had never received antidepressant medications (ρ = −0.30, *P* < 1 × 10^−26^) and after regressing out age, educational level, and sex effects (ρ = −0.29, *P* < 1 × 10^−37^). Anhedonia (BDI-II anhedonia subscale) did not correlate with the emotional impact of RPEs (ρ = 0.04, *P* = .13), but the correlation for the remaining BDI-II questions remained significant (ρ = 0.06, *P* = .01). Anhedonia was significantly correlated with baseline mood parameters (ρ = −0.25, *P* < 1 × 10^−26^) to a similar degree as the remaining BDI-II questions (ρ = −0.30, *P* < 1 × 10^−39^).

## Discussion

In this study, we provide evidence inconsistent with predictions derived from previous depression studies.^7^ Using a combination of fMRI, computational modeling, and smartphone-based data collection, we found no evidence for impairment in basic reward-related neural and emotional processes in depression in a nonlearning context. Our results suggest that the dopaminergic RPE signal is not fundamentally affected by depression. Prior observations might be best interpreted as reflecting changes in the dopaminergic effect on downstream targets, rather than a core deficit in the computation or expression of a dopaminergic RPE signal itself.

Ventral striatal BOLD activity reflects RPE signals both in reinforcement learning tasks^8^ and in gambling tasks without a significant learning requirement,^19^ where dopamine levels are known to represent RPEs.^13^ Because individuals with depression have tended to show performance deficits in complex tasks, it is important to also use paradigms where performance of depressed persons is carefully matched with that of controls. Our paradigm allowed us to evaluate depressed participants in a nonlearning task with the same level of performance (97%) as controls. The RPE signals in ventral striatum were, if anything, larger in depressed than control participants, and our sample size was larger than in previous studies reporting attenuated striatal signals in reinforcement learning tasks.^8^ Behavioral data from laboratory (n = 74) and smartphone (n = 1833) samples confirmed that depressive symptoms were not associated with a reduction in the emotional impact of RPEs.

Antidepressant drugs have a wide range of molecular targets, including receptors for neurotransmitters, such as serotonin, dopamine, norepinephrine, and glutamate. Different antidepressant drugs act at different time scales, with glutamatergic antidepressants (eg, ketamine) having more rapid effects than drugs that primarily target, for example, serotoninergic neurotransmission (eg, citalopram). The slow time constant of the latter might reflect an accumulation in the impact of altered emotional processing.^37^ Antidepressant drugs that affect dopamine transmission may have a different mechanism of action. Our results suggest that, in a nonlearning context, RPEs retain their effect on ventral striatal activity, suggesting that computation of dopaminergic RPEs is not affected by moderate depression. This leaves open the possibility that an antidepressant efficacy of dopaminergic drugs might derive from effects on downstream targets that modulate belief updating and associated adaptive behavior.

The finding of intact dopaminergic RPEs in depression is inconsistent with influential proposals,^4^ based partly on anomalous BOLD activity in the striatum. Striatal activity is likely to be modulated by many factors in addition to dopaminergic inputs. Our results support a hypothesis advanced in a recent theoretical analysis of the depression literature,^38^ where the authors proposed that depression is primarily a disorder of goal-directed decision making that relies on model-based reasoning. This type of reasoning depends on an evaluation of the environment based on a model of the causal structure of the world and may not rely substantially on dopaminergic RPEs. Dopamine’s central role in animal depression models^4^ arises out of observations that dopamine manipulations lead to depression-like behaviors, but this finding does not necessarily indicate that dopamine plays a central role in MDD. Indeed, results from human studies are equivocal, and reports of attenuated ventral striatal signals in reinforcement learning tasks^8^ may reflect impaired model-based valuation related to a mistaken understanding of the environment rather than a fundamental failure of the dopaminergic computation of RPEs. Our results support this theoretical analysis since, using a simple task that should strongly drive dopaminergic RPEs and that minimizes the likelihood of a mistaken understanding of the environment, we found no evidence for attenuated RPE signals in ventral striatum.

### Limitations

A limitation of the present study is that the majority of participants with depression were receiving antidepressant drugs. It is possible that these drugs affected neural or emotional responses to RPEs. However, we saw no difference in neural signals in medicated and medication-free depressed participants, although the medication-free group was small (n = 9). We also saw no differences in the smartphone data in individuals who had never received antidepressant medications. When we examined whether anhedonia explained changes in mood parameters, we found no evidence that this symptom related more closely to mood effects than other symptoms. We also found no association between anhedonia and ventral striatal RPE signals. We found no indication that moderate depression reduced RPE signals in ventral striatum and that mild-to-severe depressive symptoms are associated with a reduced emotional impact of RPEs in laboratory or smartphone samples. It remains possible that individuals with severe depression have attenuated ventral striatal RPE signals, although such persons will often have other major psychiatric conditions that could act as contributing factors.

## Conclusions

By using the computational modeling approach, as advocated in computational psychiatry,^40^ we show that momentary mood fluctuations in participants with depression can be predicted from the history of expectations and prediction errors resulting from those expectations. Since dopamine is known to affect the association between rewards and momentary mood,^24^ these results also support the idea that dopaminergic RPEs are intact in depression. Results from previous laboratory studies in participants without depression identified baseline mood parameters around the midpoint of the scale,^23^ suggesting that participants might center their ratings. Herein, we report that baseline mood parameters were correlated with depressive symptoms. This finding suggests that our assessment of momentary mood during a cognitive task reflects not only the impact of task events, but also an ongoing or persistent affective state that can be measured with clinical questionnaires collected on separate days. Computational modeling of momentary mood dynamics may provide a useful tool that can be associated with changes in depressive symptoms over time in future longitudinal research. Similar results in laboratory and smartphone samples demonstrate the potential for computational modeling of subjective states in psychiatric populations as well as the use of smartphones for large-scale computational phenotyping in mood disorders.

## Notes

Supplement. eMethods. Supplementary Methods **eResults.** Supplementary Results **eTable.** Characteristics of Smartphone Sample **eFigure 1.** Task Design for Neuroimaging Experiment **eFigure 2.** Task Design for Behavioral and Smartphone Experiments
