# Supplementary material for: Association of Neural and Emotional Impacts of Reward Prediction Errors With Major Depression
Source: JAMA Psychiatry. 2017 Aug 2;74(8):790–7. doi: 10.1001/jamapsychiatry.2017.1713 (PMC5710549; doi:10.1001/jamapsychiatry.2017.1713)
Supplement: Supplement. — eMethods. Supplementary Methods eResults. Supplementary Results eTable. Characteristics of Smartphone Sample eFigure 1. Task Design for Neuroimaging Experiment eFigure 2. Task Design for Behavioral and Smartphone Experiments [file jamapsychiatry-74-790-s001.pdf]

Supplementary Online Content

Rutledge RB, Moutoussis M, Smittenaar P, et al. Association of neural and emotional impacts of reward prediction errors with major depression. *JAMA Psychiatry*. Published online July 5, 2017. doi:10.1001/jamapsychiatry.2017.1713

eMethods. Supplementary Methods

eResults. Supplementary Results

eTable. Characteristics of Smartphone Sample

eFigure 1. Task Design for Neuroimaging Experiment

eFigure 2. Task Design for Behavioral and Smartphone Experiments

This supplementary material has been provided by the authors to give readers additional information about their work.

© 2017 Rutledge et al. *JAMA Psychiatry*.

eMethods. Supplementary Methods

Smartphone Sample

Participants were recruited through social media and traditional media about the project. Participants indicated their gender, level of education, and age (binned 18-24, 25-29, 30-39, 40-49, 50-59, 60-69, 70+). Participants were asked if they would be interested in contributing to depression research by completing the second edition of the Beck Depression Inventory (BDI-II) questionnaire on their smartphones. Participants with a history of depression (n=543) were more likely to be female, older than 30, not to have a university degree, and to be currently taking or to have previously taken antidepressants (eTable). In addition to the BDI-II questionnaire, we asked the following questions: Have you ever been diagnosed with depression? Have you ever taken any anti-depressant medications? If you suffer from depression, how many depressive episodes have you had in your life? If you suffer from depression, how many years of your life in total have you been depressed? Has anyone in your immediate family suffered from depression?

Probabilistic Reward Task

In each trial, subjects made a choice between the 'observation lottery' and a simultaneously presented 'decoy lottery'. Each lottery was a gamble with one or two possible outcomes (£1, £0, -£1, -£2) displayed as a pie chart where the area of each slice represented the probability of each outcome. The four observation lotteries always resulted in gaining or losing £1 and had probabilities of the better outcome of 0%, 25%, 75%, or 100%. To ensure that subjects chose the observation lottery, the decoy lottery always had a lower expected value (e.g., 75% probability of losing £1 and 25% probability of losing £2), and would result in lower earnings on average. In each trial, participants had 3 seconds to press a button corresponding to their choice. After a 5-second delay, the outcome of the chosen lottery was revealed for 1 second. Participants completed instructions and a practice session before completing 164 trials in the magnetic resonance imaging scanner. All outcomes counted for real money but participants were not aware of their earnings until completion of all tasks.

Functional Magnetic Resonance Imaging

Each volume contained 43 slices of 3-mm isotropic data (echo time of 30 ms, repetition time of 3.01 s, slice tilt of -30 degrees, Z-shim of -0.4 mT/m ms, ascending slice acquisition order). To account for T1 saturation effects, the first 5 volumes of each scan were discarded. Physiological monitoring included measurements of pulse and breathing using the Spike2 data acquisition system (Cambridge Electronic Design Limited, Cambridge, England). Field maps were acquired to allow for subsequent geometric distortion correction and T1-weighted images were acquired for structural alignment. Preprocessing followed standard procedures (EPI unwarping using field maps, slice-time correction to the first volume, motion correction, spatial transformation to the Montreal Neurological Institute (MNI) template, spatial smoothing with a Gaussian kernel of 8-mm full-width at half-maximum).

General Linear Model (GLM) analysis included stick function regressors for event onsets and parametric regressors for the expected value of chosen gambles and the predicted reward prediction error (RPE) for outcomes. Outcome regressors were lagged by 1 second due to the non-specific early peak present in striatal RPEs,[1] a signal also present in phasic dopamine concentrations.[2] To verify that RPE signals reflected both rewards and expectations, we split the RPE term into separate regressors for reward magnitude and gamble expected value (EV).[3] We included both regressors in the GLM without orthogonalization, to allow them to compete for variance. The ventral striatum region of interest was defined as 6-mm spheres at MNI coordinates (left hemisphere: -10, 12, -8; right hemisphere, 10, 12, -8) from prior studies.[4,5] The GLM also included 18 cardiac and respiratory regressors to correct for physiological noise and 6 regressors to correct for motion-induced noise.

Bayesian Model Comparison for Momentary Mood Model

We compared our RPE model for happiness to two additional models using Bayesian model comparison, which penalizes for model complexity, and where the model with the lowest Bayesian Information Criterion (BIC) score is preferred. We z-scored ratings to prevent individuals with greater rating variance from having a disproportionate influence on the model comparison. The constant term is omitted in such fits and so the RPE model has 4 parameters. All models include parameters for certain rewards and a forgetting factor. The Reward model has 3 parameters and includes a gamble reward parameter and no expectation parameter. The Reward and Expectation model has 5 parameters and includes parameters for EV of chosen gambles, gamble rewards, and the EV of gamble outcomes.

© 2017 Rutledge et al. *JAMA Psychiatry*.

eResults. Supplementary Results

Momentary Mood Model Results for Laboratory Sample

For the laboratory sample (n = 74), the RPE model for happiness was preferred (lower BIC) by Bayesian model comparison (BIC, -1063) to a simpler model that omits expectations (BIC, -727) and a more complicated model that splits the RPE term into gamble reward and gamble EV (BIC, -926). This strongly supports the idea that happiness depends on the history of expectations and RPEs resulting from those expectations. Because outcomes were shown in only half of trials in the laboratory sample, we can estimate the differential impact of expectations at the time of choices and outcomes. We found, as predicted, that choice expectation parameters were positive ($z = 3.85$, $P < .001$), reward magnitude parameters were positive ($z = 5.65$, $P < 1 \times 10^{-7}$), and outcome expectation parameters were negative ($z = -4.83$, $P < 1 \times 10^{-5}$).

eReferences

1. Rutledge RB, Dean M, Caplin A, Glimcher PW. Testing the reward prediction error hypothesis with an axiomatic model. *J Neurosci*. 2010;30(40):13525-13536.
2. Hart AS, Rutledge RB, Glimcher PW, Phillips PEM. Phasic dopamine release in the rat nucleus accumbens symmetrically encodes a reward prediction error term. *J Neurosci*. 2014;34(3):698-704.
3. Behrens TEJ, Hunt LT, Woolrich MW, Rushworth MFS. Associative learning of social value. *Nature*. 2008;456(7219):245-249.
4. Pessiglione M, Seymour B, Flandin G, Dolan RJ, Frith CD. Dopamine-dependent prediction errors underpin reward-seeking behaviour in humans. *Nature*. 2006;442(7106):1042-1045.
5. Rutledge RB, Skandali N, Dayan P, Dolan RJ. A computational and neural model of momentary subjective well-being. *Proc Natl Acad Sci U S A*. 2014;111(33):12252-12257.

© 2017 Rutledge et al. *JAMA Psychiatry*.

Characteristic	Past depression diagnosis (n = 543)	No past depression diagnosis (n = 1290)
Female, No. (%)	343 (63)	575 (45)
Aged younger than 30 y, No. (%)	130 (24)	463 (36)
University degree, No. (%)	322 (59)	880 (68)
Current medication, No. (%)	167 (31)	9 (1)
Previous medication, No. (%)	282 (52)	58 (4)
Past episodes (at least 2), No. (%)	367 (67)	88 (7)
Duration (at least 1 year), No. (%)	424 (78)	88 (7)
Family history, No. (%)	350 (64)	537 (42)
BDI-II score, mean (SD)	17.1 (12.7)	10.4 (9.3)
BDI-II anhedonia score, mean (SD)	3.7 (2.8)	2.4 (2.2)

eTable. Characteristics of Smartphone Sample

BDI questionnaires were sent to all users. Of the users that completed the questionnaire, 1833 had previously completed the game ‘What makes me happy?’ and also identified as having been diagnosed (n = 543) or never diagnosed (n = 1290) with depression. The high fraction of users with a history of depression likely stems from the way participants were invited to complete the questionnaire; app users were asked if they had time to contribute to our research on depression. Subjects who self-identified as having been previously diagnosed with depression had higher BDI scores and were more likely to have taken antidepressant medications.

© 2017 Rutledge et al. *JAMA Psychiatry*.

eFigure 1. Task Design for Neuroimaging Experiment

In each trial, participants had to select one of two lotteries with slice area representing outcome probability. There were four observation lotteries with outcomes of gaining or losing £1 and a 0%, 25%, 75%, or 100% probability of the better outcome. The task design was such that participants should always choose the observation lottery. After a 5s delay, the outcome was revealed.

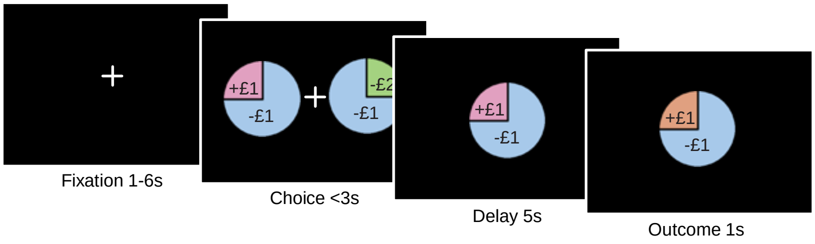

© 2017 Rutledge et al. *JAMA Psychiatry*.

eFigure 2. Task Design for Behavioral and Smartphone Experiments

A, In the laboratory experiment, participants played a risky decision task and made a choice on every trial between safe and risky options, and then were shown the outcome after a brief delay. After every 2-3 trials, participants were asked, 'How happy are you at this moment?' B, In the smartphone experiment, participants played a similar risky decision task on their smartphones. C, The momentary mood computational model accounted for happiness ratings in both depressed and control groups in the laboratory experiment.

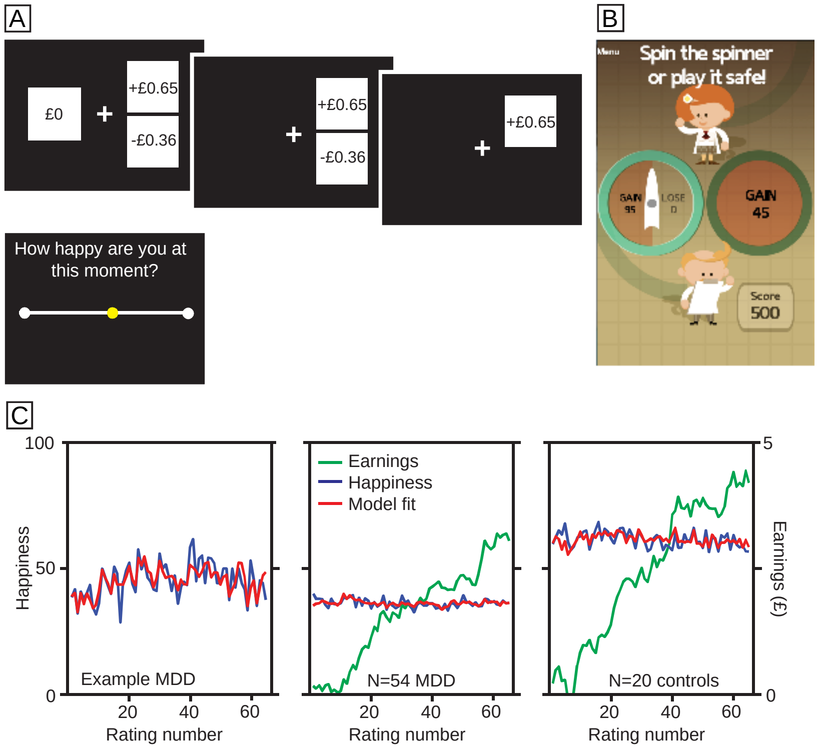

© 2017 Rutledge et al. *JAMA Psychiatry*.
